# Locus of Control and Mental Health: Human Variation Complicates a Well‐Established Research Finding

**DOI:** 10.1002/ajhb.24147

**Published:** 2024-08-14

**Authors:** Bonnie N. Kaiser

**Affiliations:** ^1^ Department of Anthropology, Global Health Program University of California San Diego La Jolla California USA

**Keywords:** daily stressors, Haiti, locus of control, mental health, sent spirit

## Abstract

**Introduction:**

Locus of control (LoC) refers to one's expectation that life outcomes and (mis)fortune are driven largely by one's own actions or abilities (internal LoC) or by external factors (e.g., powerful others, chance; external LoC). There is a large literature demonstrating an association between internal LoC and positive mental health outcomes. However, this research is conducted mostly in high‐income, Global North settings, with limited consideration of cross‐cultural variability. This short report explores how LoC relates to mental health when considered in a less‐studied context: in a setting of stark structural violence and in relation to supernatural agents.

**Methods:**

I conducted a community‐based survey in rural Haiti (*n* = 322) that assessed sent spirit‐related locus of control (LoC‐S) and mental health.

**Results:**

Among individuals experiencing higher levels of daily stressors, depressive and anxiety symptoms were high regardless of LoC‐S. However, for individuals facing low‐to‐moderate daily stressors, external LoC‐S (believing one does not have control in relation to sent spirits) was associated with lower depressive and anxiety symptoms, though this interaction did not hold for anxiety after controlling for covariates. Though initially a nonintuitive finding, I contextualize this outcome in relation to ethnographic work in Haiti, showing that the ability to explain misfortune via the supernatural world can serve as a form of blame displacement.

**Conclusion:**

In a context where extreme structural violence means that individuals realistically have little control over their lives, an external LoC better reflects lived experience, helping explain the association with better mental health outcomes.

## 1 Introduction

Since the concept of locus of control (LoC) was introduced over 50 years ago, it has become one of the most studied and influential concepts in psychology, generating over 4000 articles and 20 000 citations (Infurna and Reich 2017). In most of this research, internal LoC is considered positive, while external LoC is associated with disorders like depression, anxiety, and obsessive‐compulsive disorder (Rubenstein, Alloy, and Abramson 2017).

However, there are several shortcomings of this research. Like most psychology research, the vast majority of LoC studies have been conducted on WEIRD (Western, Educated, Industrialized, Rich, Democratic) samples (Henrich, Heine, and Norenzayan 2010; Infurna and Reich 2017). Expanding LoC research beyond WEIRD contexts would improve our understanding of the concept. First, most research on LoC reflects assumptions about individuality, choice, and agency that are specific to WEIRD contexts and conceptualizations of the self, like white, middle‐class US cultural values (Stephens, Fryberg, and Markus 2011). Similarly, there is a need to consider mismatches between perceived control and environment (Hall et al. 2010; Infurna and Reich 2017). Gurin, Gurin, and Morrison (1978) argue that greater external LoC among minoritized groups reflects “a *correct perception* of a harsh environment over which they had little control” (p. 292, emphasis added). Finally, there is a need to expand our consideration of what external LoC means—in other words, what it is attributing agency to. Typically, loci considered include powerful others, government/social systems, fate, luck, and nature, which are particularly salient in WEIRD contexts (Galvin et al. 2018). Fewer studies have explored nonhuman agents like God/s and supernatural phenomena like the paranormal (e.g., Holt, Clark, and Klem 2007; Randall and Desrosiers 1980). Research examining LoC in relation to supernatural agents will help to expand the concept beyond WEIRD contexts to provide a more complete cross‐cultural picture of how external LoC might be conceptualized.

This short report explores how LoC in relation to supernatural agents is associated with mental distress in a setting marked by limited agency. Haiti is a context where an internal LoC is much less of a “correct perception” of one's environment than in WEIRD contexts. Despite being the first independent Black republic, Haiti has experienced a deep history of international intervention and control—from slavery to US military occupation to controversial UN missions and numerous international NGOs operating almost entirely unregulated. This history has largely produced today's entrenched poverty and striking class divides, contributing to a very real experience of lack of agency (Casimir 2020; Trouillot 1995). Additionally, Haiti has experienced numerous devastating natural disasters, including the 2010 earthquake, and political crises sometimes marked by extreme violence (James 2010).

In Haiti, interactions with the supernatural world are seen as common place yet diverse. An extensive literature details Haitian concepts of the supernatural world and the multiple ways that benefits can be bestowed and harm can be wrought (e.g., Brodwin 1996; Métraux 1972; Vonarx 2007). Although the existence of supernatural agents is taken for granted, there is variability in perceived susceptibility or control in relation to them. This context thus provides fertile ground to explore LoC in relation to supernatural agents.

## 2 Methods

As part of a broader study of mental health experiences (Kaiser 2015), I locally developed a measure of LoC in relation to sent spirits (LoC‐S). Rather than supernatural “beliefs,” the LoC‐S scale assesses perceived level of personal susceptibility to a specific form of supernatural harm. Drawing on 3 months of exploratory data collection, including formal and informal interviews (*n* = 52) and participant observation, I identified central elements of supernatural relations and perceptions regarding what level of control is possible. Particularly relevant were interviews and participant observation with hougan (Vodou priests), people seeking their services, and others who had experienced sent spirits, a particular form of supernatural harm initiated by humans and performed by a boko (sorcerer). I developed a set of statements (e.g., “I do not have the capacity to protect myself from bad spirits”) to assess participants' agreement, from “Strongly agree” to “Strongly disagree.” I worked with five Haitian research assistants to pilot the LoC‐S scale (*n* = 62), with a subset of participants (*n* = 13) asked to explain how they understood what the items were asking. Scale items were adjusted when comments indicated that items were not well understood or were redundant with other items, and items were removed when pilot quantitative responses were so consistent as to suggest culturally expected “right” answers (see Table 1). There was originally an additional subscale of items related to God, but approximately 90% of participants endorsed an external LoC for these items, so the final scale focused only on sent spirits.

**TABLE 1:** Items piloted for locus of control‐sent spirits (LoC‐S) scale.

**Items focused on sent spirits**
I will know/feel if someone tries to persecute me [send a bad spirit to me]
Just like protecting myself from illness, I can protect myself from bad spirits
Even if I engage in good behaviors, *kout zonbi* [a form of sent spirit] can happen to me
I do not have the capacity to protect myself from bad spirits
*Items removed due to too little variability in responses*
If someone wanted to exchange me to an *hougan*, they could do so easily without me realizing it
I can avoid bad things happening if I serve the spirits well
I don't want to advance (in society) too much because people will do harm to me
**Items focused on God—all items cut due to too high agreement**
Prayer is not the most important thing I can do to stay healthy
There is no point in trying to fix problems myself when it's all up to God anyway
I can turn my problems over to God and know fully that he will take care of them
God will not take care of me if I do not take care of myself

In April–May 2013, we conducted a community‐based survey (*n* = 322) that included the LoC‐S scale and existing mental health screeners: a locally developed and validated depression screening tool (the Zanmi Lasante Depression Symptom Inventory (ZLDSI); Rasmussen et al. 2015) and a culturally adapted Beck Anxiety Inventory (BAI; Kaiser et al. 2013). The survey also included other locally developed tools, including a measure of 14 daily stressors (e.g., overcrowding, lack of food, flooding). The study was approved by Emory University IRB, and all participants provided informed consent.

Responses on the LoC‐S, ZLDSI, BAI, and daily stressor scale were summed to yield an overall score, using mean imputation to account for missingness, with potential scores ranging from –6 to 6 (LoC‐S), 0 to 39 (ZLDSI), 0 to 60 (BAI), and 0 to 14 (daily stressors). Items on the LoC‐S measure were reverse‐scored as relevant, so that positive scores reflected external LoC. For visualization, participants were dichotomized into internal or external LoC‐S based on mean scores indicating average (dis)agreement with items. Pearson correlations were used to assess bivariate associations between the LoC‐S scale and other study variables and scales. Linear regression was then used to model associations between continuous daily stressor and LoC‐S scores with mental health scales.

## 3 Results

**FIGURE 1:**
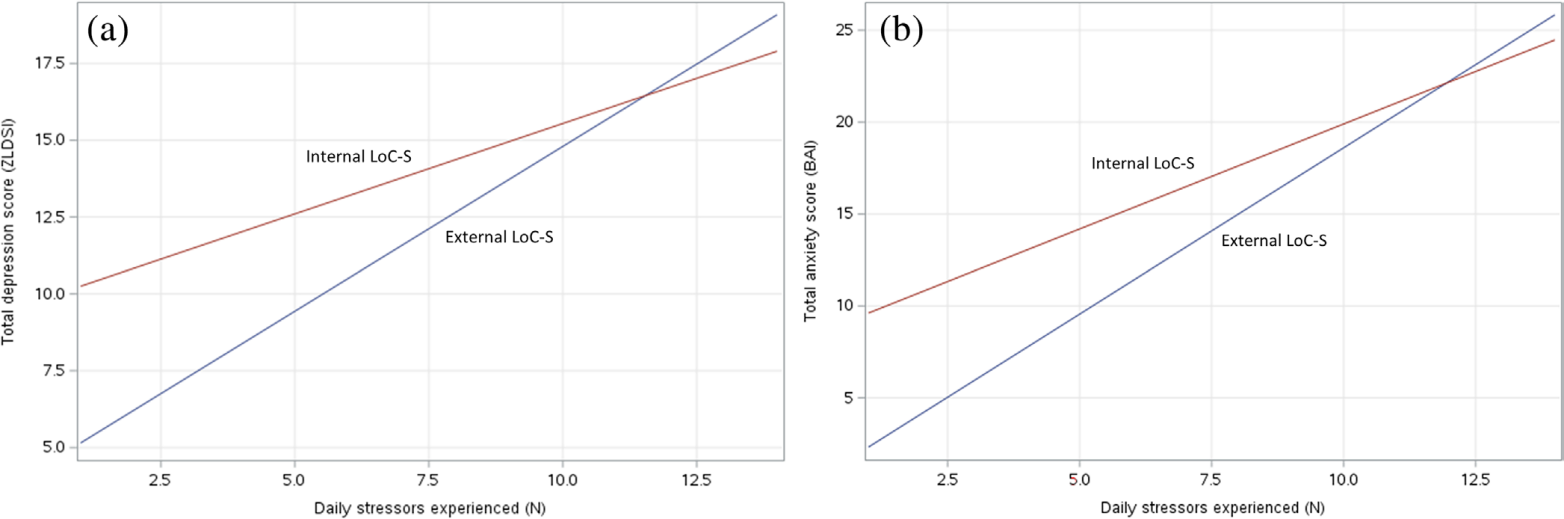
Interaction between daily stressors scale and sent spirit‐related locus of control in predicting (a) depressive and (b) anxiety symptom scores (*n* = 305). LoC‐S, locus of control‐sent spirits.

Most scales had high internal consistency (Table 2); the LoC‐S scale *α* = 0.63, comparable to other short LoC scales. Among survey participants, there was a high degree of unemployment and daily stressors and low levels of education (Table 2). Mean ZLDSI scores indicated that over half of participants met the cut‐off for mild depression and would benefit from further mental health evaluation. There was a statistically significant interaction between daily stressors and LoC‐S: among those experiencing more daily stressors, mean depressive and anxiety symptoms were high regardless; at low‐to‐moderate daily stressors, external LoC‐S was associated with lower depressive and anxiety symptoms (*p* < 0.05; Figure 1). When controlling for covariates, LoC‐S and daily stressors were both significant predictors of depressive symptoms, and the interaction approached significance (*p* = 0.055), whereas only daily stressors remained a significant predictor of anxiety symptoms (Table 3).

**TABLE 2:** Demographic and mental health characteristics of survey participants (*n* = 322).

Variable	*n* (%) or mean (range)	Correlation with LoC‐S (*r*)	Cronbach's *α* for scale
Demographics			
Age	39 (18–85)	−0.01	
Gender = female	160 (50%)	−0.32***	
Married	231 (72%)	−0.06	
Number of children	3.7 (0–14)	0.10	
Socioeconomic status			
Education (years)	3 (0–15)	0.11	
No formal employment	164 (51%)	−0.27***	
Outcome measures			
Locus of control‐sent spirits scale	−1.6 (−5 to 5)		0.63
External LoC‐S	109 (35%)		
Daily stressors scale	7.4 (1–14)	0.44***	0.76
ZL Depression Symptom Inventory	13.4 (0–30)	−0.04	0.75
Beck Anxiety Inventory	16.0 (0–55)	−0.01	0.88

*** *p* < 0.0001.

**TABLE 3:** Linear regression model of depressive and anxiety symptom scores (*n* = 305).

Variable	Depressive symptom score (*r* ^2^ = 0.31)		Anxiety symptom score (*r* ^2^ = 0.36)	
	Adjusted *β*	*p*	Adjusted *β*	*p*
Locus of control‐sent spirits^a^	−0.85	0.019	−0.54	0.352
Daily stressors scale	0.81	<0.0001	1.23	<0.0001
LoC‐S × daily stressors scale	0.08	0.055	0.05	0.440
Gender = female	3.37	<0.0001	7.32	<0.0001
Age (10‐year increase)	0.56	0.008	0.13	0.689
Education (years)	−0.12	0.166	−0.23	0.094
No formal employment	0.02	0.969	0.86	0.370

^a^ Higher score represents more external locus of control.

## 4 Discussion

These findings suggest that external LoC in relation to sent spirits is associated with better mental health, particularly for depressive symptoms. Elsewhere, I have used ethnographic data (Kaiser and Fils‐Aimé 2019) to argue that sent spirit narratives—explaining misfortune through reference to intentional harm wrought by others via the spirit world—can act as a form of blame displacement. In situations of misfortune where one might blame themselves, an alternate narrative is that it was due to a sent spirit, outside of one's control. As an external explanation for misfortune, sent spirit narratives could provide coherence between one's lived experience and perceived LoC. In contrast, those who explain misfortune through self‐blame could be considered to have a LoC that is dissonant with their environment—in this case, one of extreme structural violence that limits opportunities to avert misfortune. By providing an explanation external to oneself, sent spirits do the “work of culture” by making sense of misfortune while preserving a positive sense of self and avoiding harmful emotions (Hollan 1994; Lewis 2018; Obeyesekere 1990; Spiro 1952).

I join other researchers who have questioned the presumed‐universal association of internal LoC with better outcomes, including mental health (e.g., Hall et al. 2010; Infurna and Reich 2017). By considering this association in non‐WEIRD settings, we encounter contexts where external LoC better reflects lived reality. In such settings, having a LoC that is dissonant with that reality (i.e., an internal LoC) could potentially be detrimental. While the current study cannot make claims of causality, ethnographic data support the hypothesis that sent spirits provide a form of blame displacement that can protect against depressive symptoms. Anthropologists have posited that sorcery accusations like sent spirits could be either harmful or protective for wellbeing depending on context (Brison 1995; Schieffelin 1985; Wellenkamp 1988). This study provides one approach to quantitatively test such associations.

There are numerous existing LoC scales, from general to domain‐specific like health and work (Furnham and Steele 1993; Infurna and Reich 2017). This study introduces a new approach to assessing LoC by examining perceptions in relation to a particular category of supernatural agent. While most LoC scales have between one‐ and three‐dozen items, there have been calls for additional short scales like the LoC‐S (Galvin et al. 2018). Alternatively, future research could expand the preliminary LoC‐S scale to provide a more comprehensive assessment of supernatural LoC in Haiti, expanding beyond sent spirits to consider a broader set of supernatural agents and potential forms of harm and support from the supernatural world.

## 5 Conclusion

This short report provides initial support for a hypothesis that an external LoC is associated with better mental health outcomes in Haiti, a context of extreme structural violence. These findings agree with the relatively limited research about LoC that focuses on non‐WEIRD settings, by questioning the purportedly universal association between internal LoC and better mental health. More research is needed to investigate whether these findings hold true in other settings where an external LoC better reflects people's lived reality.

## Conflicts of Interest

The author declares no conflicts of interest.

## Data Availability

The data that support the findings of this study are available from the corresponding author upon reasonable request.
